# Chronic Alcohol Dysregulates Skeletal Muscle Myogenic Gene Expression after Hind Limb Immobilization in Female Rats

**DOI:** 10.3390/biom10030441

**Published:** 2020-03-12

**Authors:** Danielle E. Levitt, Alice Y. Yeh, Matthew J. Prendergast, Ronald G. Budnar, Jr., Katherine A. Adler, Garth Cook, Patricia E. Molina, Liz Simon

**Affiliations:** Department of Physiology, Louisiana State University Health Sciences Center, New Orleans, LA 70112, USA; dlevit@lsuhsc.edu (D.E.L.); ayeh1@lsuhsc.edu (A.Y.Y.); mprend@lsuhsc.edu (M.J.P.); budnarr@gmail.com (R.G.B.J.); kadler@lsuhsc.edu (K.A.A.); gwcook731@aol.com (G.C.); PMolin@lsuhsc.edu (P.E.M.)

**Keywords:** ethanol, ovariectomy, immobilization, recovery, regeneration, inflammation, ovarian hormone loss

## Abstract

Alcohol use and aging are risk factors for falls requiring immobilization and leading to skeletal muscle atrophy. Skeletal muscle regeneration is integral to post-immobilization recovery. This study aimed to elucidate the effects of alcohol and ovarian hormone loss on the expression of genes implicated in muscle regeneration. Three-month-old female rats received an ovariectomy or a sham surgery, consumed an alcohol-containing or control diet for 10 weeks, were subjected to unilateral hind limb immobilization for seven days, and finally were allowed a three (3d)- or 14 (14d)-day recovery. Immobilization decreased the quadriceps weight at 3d and 14d, and alcohol decreased the quadriceps weight at 14d in the nonimmobilized hind limb (NI). At 3d, alcohol decreased gene expression of myoblast determination protein (MyoD) in the immobilized hind limb (IMM) and myocyte enhancer factor (Mef)2C and tumor necrosis factor (TNF)α in NI, and ovariectomy increased MyoD and decreased TNFα expression in NI. At 14d, alcohol increased the gene expression of Mef2C, MyoD, TNFα, and transforming growth factor (TFG)β in IMM and decreased monocyte chemoattractant protein (MCP)1 expression in NI; ovariectomy increased TNFα expression in NI, and alcohol and ovariectomy together increased Mef2C expression in NI. Despite increased TGFβ expression, there was no concomitant alcohol-mediated increase in collagen in IMM at 14d. Overall, these data indicate that alcohol dysregulated the post-immobilization alteration in the expression of genes implicated in regeneration. Whether alcohol-mediated molecular changes correspond with post-immobilization functional alterations remains to be determined.

## 1. Introduction

Chronic alcohol use increases the risk of injuries, including falls caused by incoordination, imbalance, and motor vehicle accidents [1,2,3]. Approximately 40% of traumatic injuries involve alcohol intoxication [1,4], and falls are the most frequent [5]. Alcohol is an independent risk factor for osteoporotic fractures [6,7,8], including hip fractures [8,9,10]. Postmenopausal women are at an even greater risk for fractures [11,12] due to the increased prevalence of osteoporosis, frailty, and decreased muscle strength [13,14]. The increased rate of fall-related injuries in individuals with unhealthy alcohol use leads to a higher incidence of immobilization or prolonged bed rest, especially in the aging population. Approximately 30% of hospitalized older patients are diagnosed with alcohol use disorders [13].

Muscle disuse atrophy often accompanies immobilization and can either be generalized (e.g., resulting from extended periods of bedrest) or relatively localized (e.g., isolated limb immobilization). Reduced skeletal muscle mass following immobilization persists even after return to activity [14,15] and contributes significantly to the overall health care burden and decreased quality of life among affected individuals. 

Independent of injury or immobilization, alcoholic myopathy occurs in 40‒60% of chronic heavy alcohol users [16,17] and is one of the earliest pathological tissue changes observed with unhealthy alcohol use [18,19,20]. Although alcohol-related muscle disease is nearly five times more common than liver cirrhosis [18], mechanistic data are lacking on its contribution to long-term health in the context of aging and disuse atrophy. Evidence of alcoholic myopathy is associated with cumulative lifetime consumption of alcohol, and changes are most evident with long-term, high-dose consumption [16,17]. Importantly, alcohol during immobilization can accentuate disuse atrophy and impair recovery of muscle mass [3].

In chronic alcohol users, tumor necrosis factor (TNF)α is inversely related to lean mass [21]. Moreover, rodent studies have demonstrated that chronic alcohol feeding led to a sustained increase in TNFα and interleukin (IL)-6 mRNA [22]. Our published work shows decreased differentiation potential of cultured myoblasts isolated from skeletal muscle of chronic binge alcohol-administered male macaques. This decreased differentiation potential impairs myotube formation and is associated with reduced expression of myogenic genes (e.g., myoblast determination protein (MYOD), myogenin (MYOG), and myocyte enhancer factor 2C (MEF2C)) [23,24]. The marked dysregulation of myoblast myogenic and inflammatory gene expression and myotube formation with chronic alcohol administration reflects impaired muscle regenerative capacity and is likely to contribute to decreased muscle mass, especially in response to an injury. 

In normal muscle repair, satellite cells use the basement membrane of atrophying fibers as a scaffold for regeneration. In parallel, muscle repair requires the migration and proliferation of fibroblasts that produce increased extracellular matrix (ECM) components such as collagen that are degraded as myofibers regenerate [25]. Perturbation of any of these stages can result in impaired regeneration, characterized by persistent myofiber degeneration, inflammation, and fibrosis [26,27]. Persistent inflammation due to alcohol can also result in an excess of growth factors, cytokines, and collagen accumulation. In a non-human primate model of simian immunodeficiency virus (SIV) infection, we have previously established that chronic binge alcohol promotes a profibrotic milieu in skeletal muscle [28], suggesting that this mechanism may play a crucial role in impaired recovery from disuse atrophy. 

Despite epidemiological data indicating an increased incidence of alcohol-related falls and immobilization, no preclinical studies have elucidated the precise mechanisms of alcohol-accentuated disuse atrophy and influence of ovarian hormone loss. A single preclinical study on alcohol-accentuated disuse atrophy identified decreased protein synthesis and increased ubiquitin proteasome pathway (UPP)-mediated proteolysis as potential mechanisms during the initial period of recovery [29]. Moreover, few studies have investigated the mechanisms involved in recovery following immobilization. Given this gap in the literature, the purpose of this study was to examine the influence of alcohol and ovarian hormone loss on gene expression implicated in muscle regeneration following immobilization. Our main finding was that, in alcohol-fed animals, there was an early overall decrease followed by a later substantial increase in markers of muscle regeneration post-immobilization.

## 2. Materials and Methods

### 2.1. Animal Study Design

All experiments were approved by the Institutional Animal Care and Use Committee (IACUC #3477, last approved 8/10/2018) at Louisiana State University Health Sciences Center (LSUHSC) in New Orleans, LA, and adhered to the National Institutes of Health guidelines for the care and use of experimental animals. Forty three-month-old F344 (CDF) female rats (Charles River) were singly housed during a week-long acclimation period in a controlled environment and for the entire study period.

### 2.2. Ovariectomy

Animals were randomly assigned to ovariectomy (OVX) or sham surgery (SHAM). Animals were anesthetized using ketamine/xylazine and were given a dose of buprenorphine SR before surgery. A bilateral 0.5 cm skin incision, 1.5‒2.0 cm lateral from the spine with its cranial terminus 1.5‒2.0 cm from the 13th rib was made, followed by muscle incision. The ovaries located in the fat pad were drawn out using blunt forceps. Ovarian blood vessels were ligated with 4-0 silk and the ovaries were excised. The muscle wall and skin incisions were sutured separately. Animals were allowed to recover for three days. Sham surgery was performed similarly, except the ovaries were not clamped or excised. Uterine weights measured at end point were used to indicate effectiveness of ovarian hormone loss. The mean uterine weight was 0.11 ± 0.01 g in the OVX group, which was significantly lower than the mean uterine weight in the SHAM group (0.28 ± 0.03 g). 

### 2.3. Alcohol Feeding

One week after surgery, half of the animals in the OVX and SHAM groups were assigned to consume an alcohol-containing diet (ALC) and the other half a pair-fed control diet (VEH) for 10 weeks. Animals were transitioned into a Lieber‒DeCarli liquid diet (Bio-Serv, Frenchtown, NJ, USA; ethanol diet F1258SP and control diet F1259SP) by increasing the liquid diet and decreasing solid food over five days. Rats in the ALC group received 36% of their calories from alcohol; those in the VEH group were isocalorically matched. Animals were weighed once per week and blood alcohol concentrations were measured at the end of the animal’s dark cycle. The average blood alcohol concentration in the ALC group was 0.1 g/dL. Blood was collected by snipping the tail tip and collecting ~50 µL of blood after five weeks of alcohol consumption, on the days of cast application and removal, and on the day of euthanasia. The liquid diet was available to the animals until the time of tail bleeding. 

### 2.4. Unilateral Hind Limb Immobilization

**Figure 1:**
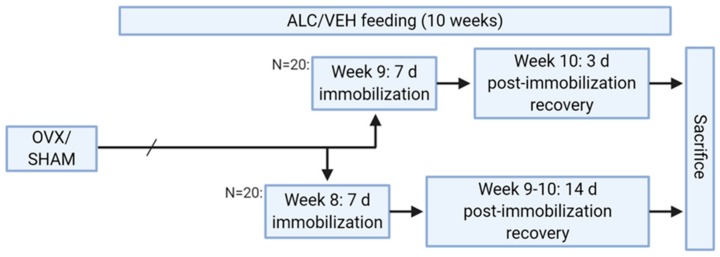
Schematic of study design. OVX: ovariectomy, SHAM: sham surgery, ALC: alcohol-containing diet, VEH: control diet.

Animals were anesthetized with ketamine/xylazine and unilateral hind limb immobilization performed using plaster of Paris. Animals were casted with slight hip flexion and knee extension to allow for quadriceps muscle atrophy. The casts were not placed on the dorsal aspect of the ankle or on the feet, and this method of casting does not prevent movement or obstruct blood flow. The animals were immobilized for seven days either beginning at week 8 and allowed to recover for 14 days or beginning at week 9 and allowed to recover for three days (Figure 1). 

This method of hind limb immobilization was reported to achieve quadriceps muscle atrophy [30], and the seven-day immobilization period was chosen because previous time-course studies demonstrated that seven days post-immobilization, gastrocnemius weight reached the lowest value and molecular markers of atrophy and early markers of muscle regeneration were increased [31,32]. After either three or 14 days of post-immobilization recovery, body weight was measured, and rats were euthanized by anesthetic overdose (ketamine/xylazine, 100/10 mg/kg administered intraperitoneally) prior to decapitation. Previous studies report that muscle atrophy remains after three days [31], but is recovered by 14 days post-immobilization or post-hind limb unloading in healthy animals, but not in those with ovarian hormone loss [33,34] or chronic alcohol [3]. Quadriceps muscles from immobilized (IMM) and nonimmobilized (NI) hind limbs were excised, weighed, and expressed relative to tibia length. The excised muscles were then snap frozen and cryopreserved at −80 °C. 

### 2.5. RNA Isolation and qPCR

RNA was extracted from 30 mg of using miRNeasy Mini Kit (Qiagen, Germantown, MD, USA). cDNA was synthesized using the QuantiTect Reverse Transcription Kit (Qiagen) and qPCR performed using RT^2^ SYBR^®^ Green Fluorescent qPCR Master mix (Qiagen) and specific primers (Integrated DNA Technologies, Coralville, IA, USA). Rps13 was used as the endogenous control [35] and values are expressed as the relative fold change using the ∆∆Ct method. All primer sequences are included in Table 1.

### 2.6. Picrosirius Staining for Collagen Expression 

Cryosections of vastus lateralis muscle from the immobilized limb after 14 days of post-immobilization recovery were stained for collagen expression using picrosirius red (PSR) staining, as described previously [28]. Fifteen-micrometer sections of muscle were fixed in ice-cold acetone, washed in PBS, and stained in Bouin’s reagent (Sigma-Aldrich, St. Louis, MO, USA) for 30 min. The sections were then rinsed in water, stained with PSR (Sigma Direct Red 80, Sigma-Aldrich) for 1 h, and rinsed in acidic water (5 mL glacial acetic acid in 1 L of water) and a picric alcohol (10% picric acid, 20% ethanol) rinse. The slides were then cleared in xylene and mounted with Permount medium (Sigma-Aldrich). Slides were imaged in a blinded manner using an Olympus DP72 Digital Camera System mounted to an Olympus BX51 TRF Microscope (Olympus, Center Valley, PA, USA), and the collagen fiber area was determined using custom image analysis software (programmed by RGB; full code can be accessed at https://github.com/rbudnar/PSR). 

### 2.7. Statistical Analyses

All data are presented as mean ± SE, where *N* = 4‒5 for each treatment group at the study endpoint. The normality of distribution and homogeneity of variance were verified using SPSS version 25 (IBM, Armonk, NY, USA). The body weight of all animals at baseline and pre-immobilization, body weight at necropsy (three days and 14 days post-immobilization), quadriceps mass ratio (immobilized versus nonimmobilized hind limb), and collagen expression were analyzed using two-way analysis of variance (ANOVA; alcohol × ovariectomy). For quadriceps weight, a paired-samples *t*-test was run to detect differences due to immobilization. Then, for quadriceps weight and gene expression data, two-way ANOVA (alcohol × ovariectomy) was used to dissect the effects of each treatment and their interaction for the nonimmobilized and immobilized hind limbs separately. Fisher’s LSD post hoc test was used to identify the location of any significant interaction effects. Statistical significance was established at *p* < 0.05. 

## 3. Results

### 3.1. Body and Quadriceps Weight

**Figure 2:**
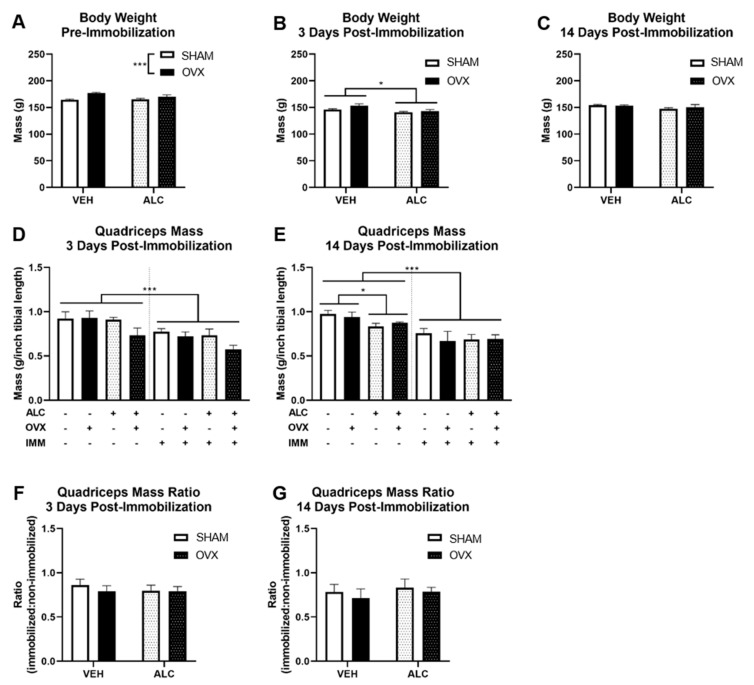
Total body weight before immobilization (**A**) and after 3 (**B**) and 14 (**C**) days of post-immobilization recovery; quadriceps mass relative to tibia length after 3 (**D**) and 14 (**E**) days of post-immobilization recovery, and ratio of immobilized to nonimmobilized quadriceps mass after three (**F**) and 14 (**G**) days of post-immobilization recovery. Body weight shown at pre-immobilization includes all animals; all other figures depict the three- or 14-day post-immobilization recovery groups. Body weights and quadriceps mass ratios were analyzed using two-way ANOVA (alcohol × ovariectomy). A paired-samples *t*-test was used to detect differences due to immobilization for quadriceps weight relative to tibia length. Then, treatment effects were analyzed using two-way ANOVA (alcohol × ovariectomy) for the nonimmobilized and immobilized hind limbs separately. VEH: pair-fed, ALC: alcohol-fed, SHAM: nonovariectomized, OVX: ovariectomized, IMM: immobilized. “+” indicates that it includes that treatment; “−“indicates that it does not include that treatment. *p < 0.05, ***p < 0.001. Means ± SEM. *N* = 4‒5 per group.

OVX animals weighed significantly more than SHAM animals before immobilization (*p* < 0.001; Figure 2A). After three days of post-immobilization recovery, ALC significantly decreased the body weight (*p* = 0.02, Figure 2B), but there were no significant differences in body weight after 14 days of post-immobilization recovery (Figure 2C). Immobilization significantly decreased the quadriceps muscle weight after three (*p* < 0.001; Figure 2D) and 14 (*p* < 0.001; Figure 2E) days of post-immobilization recovery. There were trends for ALC (*p* = 0.097) and OVX (*p* = 0.065) to decrease the quadriceps muscle weight after three days of post-immobilization recovery in the immobilized limb. ALC decreased the quadriceps weight in the nonimmobilized limb after 14 days of post-immobilization recovery regardless of OVX (*p* = 0.019; Figure 2E). No significant differences due to ALC or OVX were observed for the ratio of quadriceps muscle mass from the immobilized:nonimmobilized hind limb.

### 3.2. Myogenic Gene Expression

**Figure 3:**
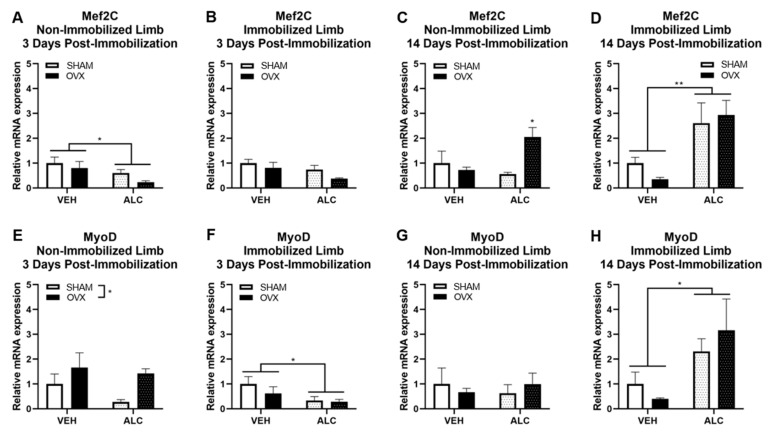
mRNA expression of myogenic genes in vastus lateralis muscle: myogenic enhancer factor 2C (Mef2C) after three days of post-immobilization recovery in the nonimmobilized (**A**) and immobilized (**B**) hind limb, Mef2C after 14 days of post-immobilization recovery in the nonimmobilized (**C**) and immobilized (**D**) hind limb, myoblast determination protein 1 (MyoD) after three days of post-immobilization recovery in the nonimmobilized (**E**) and immobilized (**F**) hind limb, and MyoD after 14 days of post-immobilization recovery in the nonimmobilized (**G**) and immobilized (**H**) hind limb with or without alcohol (ALC) and ovariectomy (OVX). Data analyzed by two-way ANOVA (alcohol × ovariectomy), *p < 0.05, **p < 0.01, Mean ± SEM.

After three days of post-immobilization recovery, ALC decreased Mef2C expression in the nonimmobilized hind limb (*p* = 0.035, Figure 3A) and nonsignificantly decreased Mef2C expression in the immobilized hind limb (*p* = 0.065, Figure 3B). After 14 days of post-immobilization recovery, there was an alcohol × ovariectomy interaction effect to increase Mef2C in the nonimmobilized hind limb (*p* = 0.025, Figure 3C), where ALC and OVX together increased Mef2C expression over all other groups. ALC increased Mef2C expression in the immobilized hind limb 14 days post-immobilization (*p* = 0.002, Figure 3D).

After three days of post-immobilization recovery, OVX significantly increased MyoD expression in the nonimmobilized hind limb (*p* = 0.046, Figure 3E), and alcohol significantly decreased MyoD expression in the immobilized hind limb (*p* = 0.047, Figure 3F). After 14 days of post-immobilization recovery, neither ALC nor OVX influenced MyoD expression in the nonimmobilized hind limb (Figure 3G), but ALC significantly increased MyoD expression in the immobilized hind limb (*p* = 0.015, Figure 3H).

Neither ALC nor OVX significantly altered myogenin or Myh1 mRNA expression after three or 14 days of post-immobilization recovery (not shown).

### 3.3. Inflammatory and Fibrotic Gene Expression

**Figure 4:**
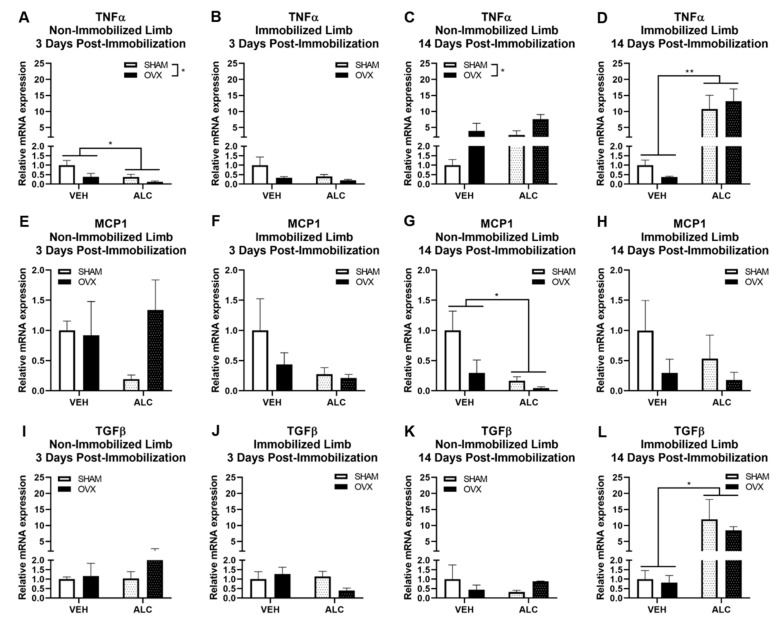
mRNA expression of inflammatory and fibrotic genes in the vastus lateralis muscle: tumor necrosis factor (TNF)α after three days of post-immobilization recovery in the nonimmobilized (**A**) and immobilized (**B**) hind limb; TNFα after 14 days of post-immobilization recovery in the nonimmobilized (**C**) and immobilized (**D**) hind limb; monocyte chemoattractant protein (MCP)1 after three days of post-immobilization recovery in the nonimmobilized (**E**) and immobilized (**F**) hind limb; MCP1 after 14 days of post-immobilization recovery in the nonimmobilized (**G**) and immobilized (**H**) hind limb; transforming growth factor (TGF)β after three days of post-immobilization recovery in the nonimmobilized (**I**) and immobilized (**J**) hind limb; and TGFβ after 14 days of post-immobilization recovery in the nonimmobilized (**K**) and immobilized (**L**) hind limb with or without alcohol (ALC) and ovariectomy (OVX). Data analyzed by two-way ANOVA (alcohol × ovariectomy), * *p* < 0.05, ** *p* < 0.01, Mean ± SEM.

After three days of post-immobilization recovery, ALC (*p* = 0.040) and OVX (*p* = 0.042) decreased TNFα expression in the nonimmobilized hind limb (Figure 4A). After 14 days of post-immobilization recovery, OVX increased TNFα expression in the nonimmobilized hind limb (*p* = 0.025, Figure 4C) and ALC increased TNFα expression in the immobilized hind limb (*p* = 0.003, Figure 4D).

Neither ALC nor OVX significantly altered MCP1 expression after three days of post-immobilization recovery (Figure 4E,F). After 14 days of post-immobilization recovery, ALC significantly decreased MCP1 expression (*p* = 0.016) and OVX nonsignificantly decreased MCP1 expression (*p* = 0.053) in the nonimmobilized hind limb (Figure 4G). Neither ALC nor OVX significantly altered MCP1 expression in the immobilized hind limb after 14 days of post-immobilization recovery (Figure 4H).

Neither ALC nor OVX significantly altered TGFβ expression after three days of post-immobilization recovery (Figure 4I,J) or after 14 days of post-immobilization recovery in the nonimmobilized hind limb (Figure 4K). ALC significantly increased TGFβ expression after 14 days of post-immobilization recovery in the immobilized hind limb (*p* = 0.012, Figure 4L).

Neither ALC nor OVX significantly altered IL-1β or IL-10 mRNA expression after three or 14 days of post-immobilization recovery (not shown).

### 3.4. Collagen Expression

**Figure 5:**
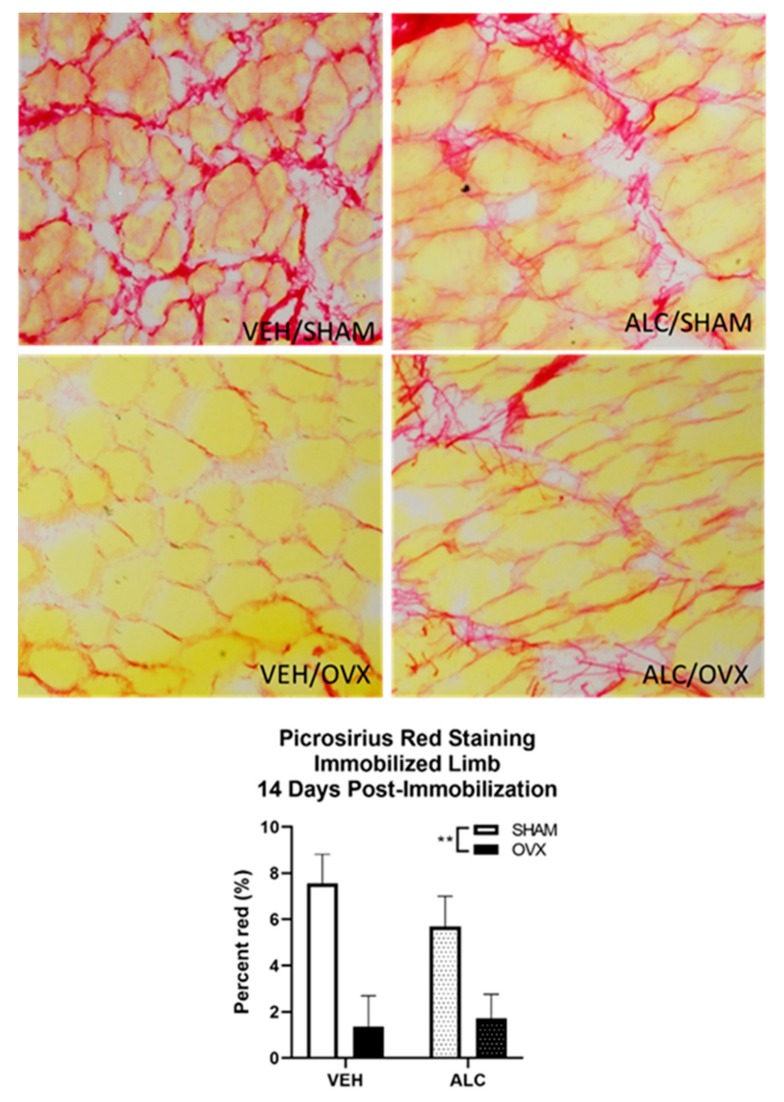
Picrosirius red staining of the quadriceps muscle of the immobilized hind limb after 14 days of post-immobilization recovery, with or without alcohol (ALC) and ovariectomy (OVX). Data analyzed by two-way ANOVA, **p < 0.01, Mean ± SEM. Images obtained at 10x magnification.

After 14 days of post-immobilization recovery, ALC did not alter collagen expression, but OVX decreased collagen expression in the immobilized limb (*p =* 0.002, Figure 5).

## 4. Discussion

In this study, the effects of chronic alcohol consumption and ovarian hormone loss on changes in the expression of genes implicated in muscle regeneration and recovery following unilateral hind limb immobilization were examined. Immobilization decreased quadriceps muscle weight after both three and 14 days of post-immobilization recovery. Alcohol decreased quadriceps weight in the nonimmobilized hind limb after 14 days of post-immobilization recovery, suggesting that alcoholic myopathy was induced in this group. Contrary to our hypothesis, quadriceps muscle weight in the immobilized hind limb was not altered by alcohol or ovariectomy. However, alcohol and ovariectomy differentially regulated important markers of muscle regeneration, with several genes increased by alcohol after 14 days of post-immobilization recovery. Moreover, effects were observed in the immobilized and nonimmobilized hind limbs, suggesting that alcohol and ovariectomy had effects on myogenic and inflammatory processes in skeletal muscle.

The regeneration of skeletal muscle involves three main phases. Initially, there is degeneration and an inflammatory response that is initiated very early and lasts for up to two weeks. The second phase, which involves regeneration, begins in the first week, and the last phase of remodeling begins in the second week, promoting revascularization and fibrosis [36]. Following atrophy or injury, MyoD, an early marker of myogenic activation and differentiation, is upregulated as early as two days post-immobilization [37]. Mef2C is a transcription factor required for early-stage myogenic function and is expressed throughout muscle differentiation [38]. Results of the present study indicate that after three days of post-immobilization recovery, alcohol decreased the expression of MyoD in the immobilized limb and Mef2C, an early marker of myogenic differentiation, in the non-immobilized limb. However, after 14 days of post-immobilization recovery, Mef2C and MyoD expression were significantly increased in the immobilized limbs, suggesting an initial suppression and late increase in the expression of markers of regeneration with alcohol. These alcohol-mediated changes in myogenic gene expression are consistent with our recent work in myoblasts, showing that in vivo chronic binge alcohol administration decreased MyoD and Mef2C expression in isolated myoblasts after seven days of differentiation in quadriceps muscles from SIV-infected rhesus macaques [23,24,39]. Furthermore, 100 mM alcohol in vitro decreased MyoD mRNA expression during 1‒3 days of differentiation in C2C12 cells [40]. In vitro findings also demonstrate that alcohol suppresses myoblast fusion under differentiation conditions [23,24,39,40]. In mice, MyoD mRNA was significantly increased after seven days of immobilization and went back to control levels by seven days of post-immobilization recovery [41]. In the present study, MyoD was decreased with alcohol at three days compared to pair-fed animals and increased after 14 days of post-immobilization recovery, suggesting that alcohol may delay a normal early increase in MyoD expression or may prolong the regeneration process such that expression in the immobilized hind limb was much higher than for pair-fed animals at two weeks post-immobilization. Moreover, ovariectomy increased MyoD expression in the nonimmobilized limb after three days of post-immobilization recovery, and alcohol and ovariectomy synergistically increased Mef2C expression in the nonimmobilized limb after 14 days of post-immobilization recovery. At this same time point, alcohol decreased quadriceps mass in the nonimmobilized limb compared to pair-fed animals, suggesting alcoholic myopathy. It is possible that the combination of alcohol feeding and ovariectomy induced more profound structural changes and that the increased Mef2C after 14 days of post-immobilization recovery in the nonimmobilized limb is a compensatory response. Taken together, the data from the current and previous studies on alcohol-mediated changes of myogenic genes suggest impaired early myogenic potential with alcohol that is not restricted to atrophied muscle.

A transient inflammatory milieu during the early repair process is required for the activation of satellite cells. MCP1, a potent chemoattractant and activating factor for macrophages, is essential for successful completion of muscle regeneration [42]. Similarly, a rapid increase in TNFα synthesis by C2C12 myoblasts during early periods of myotube differentiation is critical for muscle-specific gene expression and regeneration [43]. However, chronic inflammation can be deleterious, impairing regeneration and promoting a profibrotic milieu [44]. The present study demonstrates that after three days of post-immobilization recovery, alcohol and ovariectomy decreased the expression of the inflammatory cytokine, TNFα, in the nonimmobilized hind limb, suggesting an early decrease in TNFα that was not statistically significant for the immobilized hind limb. Moreover, alcohol increased the expression of TNFα after 14 days of post-immobilization recovery. Previous work demonstrated that 14 days of hind limb unloading increased TNFα protein expression at 1 and 5 days post-immobilization in the soleus muscle, but that the levels returned to control values at 14 days post-immobilization [45]. A separate study observed no differences in TNFα mRNA expression immediately after unloading; however, TNFα expression was not measured during the post-immobilization recovery time period [29]. Since TNFα mRNA expression was decreased after three days and drastically elevated after 14 days of post-immobilization recovery, it appears that chronic alcohol may have prevented a normal early repair process and promoted a persistent pro-inflammatory milieu. In contrast, alcohol significantly decreased MCP1 expression in the nonimmobilized hind limb after 14 days of post-immobilization recovery. MCP1 is critical to normal skeletal muscle regeneration [42]; therefore, it is noteworthy that alcohol did not significantly alter the early expression of this cytokine. Whether these molecular changes indicate an overall impairment in regeneration and whether overt alcohol-mediated changes in muscle atrophy are apparent at a later time point warrants further investigation.

In the current studies, ovariectomy altered gene expression only in the nonimmobilized hind limb. Ovariectomy reduced TNFα gene expression after three days and increased expression after 14 days of post-immobilization recovery in the nonimmobilized hind limb, suggesting an altered inflammatory milieu that was not apparent in the immobilized hind limb. Ovariectomy increased body weight prior to immobilization, but this effect was no longer observed after immobilization. Furthermore, there were no statistically significant effects of ovarian hormone loss on quadriceps weight. Previous studies have demonstrated that ovarian hormone loss prevented muscle mass recovery after 14 days of reloading following one month of hind limb unloading [33,34], but this effect of ovariectomy was ameliorated when ovariectomized animals were administered estradiol [34]. In a separate study, although 14 days of unloading decreased the mass of three of the five muscles studied at the end of the unloading period, ovariectomy did not accentuate disuse atrophy in two of the three affected muscles [46]. These data are consistent with those observed in the present study, although it is possible that ovariectomy may have accentuated atrophy in other skeletal muscles that were not examined. Whether the length of disuse atrophy or age at time of ovariectomy influences muscle changes is not known. Overall, it appears that there were no significant effects of ovarian hormone loss on myogenic or inflammatory genes in the immobilized hind limb.

In spite of an alcohol-mediated increase in TGF-β expression after 14 days of post immobilization recovery in the immobilized hind limb, there was not a corresponding increase in muscle collagen expression. Intriguingly, ovariectomy decreased collagen expression regardless of alcohol use. It is possible that the overall recovery process was impaired due to ovarian hormone loss such that neither tissue repair nor fibrosis proceeded as normal. Collagen mRNA expression is either decreased [47] or unchanged [48], and collagen content increased [49] during hind limb unloading. Heinemer et al. found that muscle collagen content was unchanged by 14 days of hind limb unloading, but significantly elevated with reloading [48]. This elevation was no longer statistically significant after 16 days of recovery, but collagen content was still numerically elevated over control animals. Studies have demonstrated that ovariectomy increases collagen content in soleus muscle in sedentary animals compared to those that were allowed to run voluntarily [50], and resistance training further increases collagen content [51]. The increase in collagen was associated with an observed increase in myosin heavy chain I content, indicating a possible fiber type shift from fast-to-slow twitch with ovariectomy [50] that was not examined in this study. The results from those studies suggest differences in contractile characteristics, with ovarian hormone loss that could be accentuated after immobilization. In contrast, the decreased collagen observed with ovariectomy in the current study may demonstrate residual atrophy of collagen fibers due to decreased load requirements during immobilization, particularly if there was an underlying fiber type shift that was not reversed with reloading.

The immobilization model is reproducible, but the extent of molecular changes varied between animals. It is also possible that differences in gene expression data are impacted by differences in the initial rate of atrophy; differences immediately post-immobilization (e.g., zero days of recovery) were not examined. Immobilization decreased the quadriceps mass by 20% and 23% after three and 14 days of post-immobilization recovery, respectively. This reduction in muscle mass is more pronounced and persistent than others have found in other muscles with hind limb immobilization. For example, after seven days of hind limb immobilization, Lang et al. observed an approximately 14% decrease in immobilized gastrocnemius after three days of post-immobilization recovery, and muscle mass was recovered by 10 days post-cast removal [52]. These differences may be due to the specific muscle under investigation. We did not observe the expected recovery of quadriceps weight by 14 days of recovery and studies are warranted to determine when there is a complete recovery of quadriceps muscle mass following immobilization.

Although this study provides valuable information about alcohol-mediated delays in myogenic gene expression, there are several limitations to the study. The sample size was relatively small, and the study would have been strengthened with a larger sample size. Whether alcohol-mediated changes would be observed at a later time point was not tested. Furthermore, our studies did not determine changes in anabolic and catabolic processes in the muscle. Previous studies have demonstrated that repeated gavages of alcohol during immobilization and for five days post-immobilization accelerated disuse atrophy by decreasing protein synthesis and increasing ubiquitin-mediated proteolysis [29]. The present model only simulated muscle atrophy following immobilization; whether alcohol impairs recovery following mechanical muscle injury or a fracture was not assessed. These studies were performed in three-month-old female rats; whether similar or exaggerated changes occur in older females or in males needs to be determined.

In summary, our data indicate that alcohol dysregulates the expression of markers of muscle regeneration following unilateral hind limb immobilization, but ovariectomy did not have an effect. Rather, ovariectomy altered gene expression in the nonimmobilized hind limb. Although alcohol and ovarian hormone loss did not significantly exacerbate the immobilization-mediated decrease in muscle weight, it is possible that underlying differences in regeneration may have occurred. Therefore, when immobilization is indicated, caution is warranted regarding alcohol use during the immobilization and post-immobilization recovery periods.

## Figures and Tables

**Table 1:** List of primers for qPCR analysis (primers from IDT Technologies).

Gene	Forward Primer	Reverse Primer
*Myod*	CAGGTGTAACCATACCC	CTGGCCAAGCAACTCTTAT
*Mef2c*	ATCTCTCCCTGCCTTCTAC	CGTGTGTTGTGGGTATCTC
*Myh1*	CCGTGAACTTGAAGGAGAAG	CCTCTTCAGTTTGGTAAG
*Myogenin*	CCACCGTCCATTCACATAAG	GGACTCCATCTTTCTCTCCT
*Mcp1*	CTCAGCCAGATGCAGTTAAT	CTGCTGGTGATTCTCTTGTAG
*Tnfa*	CGTGTTCATCCGTTCTCTAC	GAGCCACAATTCCCTTTCT
*Il-1b*	CTGACAGGCAACCACTTAC	CTGTGCACTGGTCCAAAT
*Il-10*	CGACGCTGTCATCGATTT	GGCCTTGTAGACACCTTTG
*Tgf b*	GAACCAAGGAGACGGAATAC	GGGACTGATCCCATTGATTT
*Rps13*	GCACCTTGAGAGGAACAGAA	GAGCACCCGCTTAGTCTTATAG
